# Supplementary material for: Restoring South African subtropical succulent thicket using Portulacaria afra: exploring the rooting window hypothesis
Source: PeerJ. 2023 Jul 24;11:e15538. doi: 10.7717/peerj.15538 (PMC10437031; doi:10.7717/peerj.15538)
Supplement: Supplemental Information 1 — This data highlights the effects of soil moisture and parent-plant on root inititation in Portulacaria afra cuttings. [file peerj-11-15538-s001.zip › RootingWindow_Raw data and Scripts/RootingWindow_LMMs.html]

Alastair - spekboom


# Alastair - spekboom

#### Gavin

#### 2022-08-19

# Testing for treatment effect on *Portulacaria* while accounting for originating plant

## Alastair’s comments/instructions:

Notes: 1) There could be two response variables, RootFraction or
RootMass\_g. RootFraction does take the original plant size in account,
by some degree. I am quite torn as to whether to use the raw RootMass\_g
or RootFraction. 2) The Treatment variable may or may not be significant
(it’s close to the threshold). It doesn’t really matter if it is or
isn’t. It’s the clear cut effect of the individual (Plant) that is the
most interesting. Thus, any way to demonstrate that there are plants
that poorly vs plants that performed better, overall, is the thing of
interest. I can see it in the data :) 3) The StemWidth\_mm and Height\_mm
are significantly different amongst the plants. If there is a way to
include these as predictors, and test if they are significant or not,
would be very useful. As far as I understand these things, they should
be treated as independent and not linked to plant (I think)

```
require(tidyverse)
require(nlme)
require(gsheet)
require(usdm)
require(MuMIn)
```

I recognise the value of using Groundhog to consolidate package
versions, but couldn’t get it working smoothly just yet (sorry!), so
taking the path of least resistance and starting from scratch with a new
RMD script…

## Downloading data from Google Sheet…

```
url <- 'https://docs.google.com/spreadsheets/d/14R4vNXWWqfQo9DLjalk5Qocy8p6Ceigly0-_Tt-eOdQ'
read_csv(construct_download_url(url,sheetid="260952732"))%>%
  as_tibble()->
  dat

### Data Cleanup
# Two samples lost. Have rooting points, but no root-mass...removing
# Removing unimportant columns. 
dat%>%
  filter(!is.na(RootMass_g))%>%
  filter(!(StemWidth_mm == max(StemWidth_mm[Plant=="P5"]))) %>%   #removing outlier stem width
  filter(!(Treatment=="C")) %>%                                   #removing control values
  dplyr::select(-`Cutting Symmetry`,-DegreeOfBranching,-NoOfRootingPts)->
  dat


head(dat)
```

```
## # A tibble: 6 x 11
##    Tray Column Treatment Row   Plant StemWidth_mm Height_mm RootMass_g
##   <dbl>  <dbl> <chr>     <chr> <chr>        <dbl>     <dbl>      <dbl>
## 1     1      1 D04       A     P1            5.92       220      0.135
## 2     1      1 D04       B     P5            7.05       225      0.101
## 3     1      1 D04       C     P2            5.53       212      0.036
## 4     1      1 D04       D     P7            5.94       243      0.011
## 5     1      1 D04       E     P6            5.93       229      0.038
## 6     1      1 D04       F     P3            5.04       234      0.006
## # ... with 3 more variables: StemDryMass_g <dbl>, RootFraction <dbl>,
## #   Rooted <dbl>
```

## visual data exploration

```
plot_g <- ggplot(data=dat, aes(2*pi*(StemWidth_mm/2), RootMass_g)) +
  geom_smooth() +
  geom_rug(colour = "dark grey") +
  geom_point(aes(colour = Plant)) +
  coord_cartesian(ylim = c(0,0.35))+
  facet_wrap(vars(Treatment))

plot_g
```

```
## `geom_smooth()` using method = 'loess' and formula 'y ~ x'
```

## Specify different models

```
fn <- formula(scale(RootMass_g/(2*pi*(StemWidth_mm/2))) ~ Treatment) #incorporating root mass per stem width, rather than a separate predictor

#basic linear model fitted under GLS (i.e. an ANOVA)
mod_b <- gls(fn, data = dat, method = "REML")

#model with random intercept
mod_I <- lme(fn, data = dat, method = "REML",
             random = ~1|Plant)

#model with residual variability different per plant
mod_RV <- gls(fn, data = dat, method = "REML",
              weights=varIdent(form=~1|Plant))

#model with both residual variability and random intercept
weighting <- Initialize(varIdent(form = ~1|Plant), data = dat)
mod_IandRV <- lme(fn, data = dat, method = "REML",
             random = ~1|Plant, weights = weighting)


anova(mod_I, mod_RV, mod_IandRV, mod_b)
```

```
##            Model df      AIC      BIC    logLik   Test   L.Ratio p-value
## mod_I          1  8 1335.500 1369.137 -659.7502                         
## mod_RV         2 13 1350.764 1405.424 -662.3822 1 vs 2   5.26397  0.3845
## mod_IandRV     3 14 1331.473 1390.337 -651.7365 2 vs 3  21.29129  <.0001
## mod_b          4  7 1440.463 1469.895 -713.2315 3 vs 4 122.99004  <.0001
```

```
# compares models using LRT - AIC also gives an indication (smaller the better)
```

Best fitted model includes unique variance structures at plant level
and at random intercepts (delta AIC ~2), which is “better” than the null
model and both terms separately. This suggests that there is a
plant/individual effect that needs to be accounted for. i.e. P<0.01
for LRT.

## check if terms should be removed - more parsimonious?

```
mod_ML <-  lme(fn, data = dat, method = "ML",
             random = ~1|Plant, weights = weighting)

fn2 <- update(fn, .~. -Treatment)
mod_ML_noTreat <- lme(fn2, data = dat, method = "ML",
             random = ~1|Plant, weights = weighting)


drop1(mod_ML)
```

```
## Single term deletions
## 
## Model:
## scale(RootMass_g/(2 * pi * (StemWidth_mm/2))) ~ Treatment
##           Df    AIC
## <none>       1317.0
## Treatment  5 1315.7
```

```
anova(mod_ML_noTreat, mod_ML)
```

```
##                Model df      AIC      BIC    logLik   Test  L.Ratio p-value
## mod_ML_noTreat     1  9 1315.661 1353.611 -648.8305                        
## mod_ML             2 14 1317.026 1376.059 -644.5131 1 vs 2 8.634791  0.1245
```

Check whether variables included make model more parsimonious (delta
AIC 2).

## term significance

```
fn <- update(fn, .~. )

mod_best <- lme(fn, data = dat, method = "REML",
             random = ~1|Plant, weights = weighting)

anova(mod_best)
```

```
##             numDF denDF   F-value p-value
## (Intercept)     1   489 0.0365829  0.8484
## Treatment       5   489 1.8322969  0.1050
```

Treatment is not significant overall when accounting for random
intercepts (i.e. plant differences) and residual variance per plant.

## Model output parameters

```
summary(mod_best)
```

```
## Linear mixed-effects model fit by REML
##   Data: dat 
##        AIC      BIC    logLik
##   1331.473 1390.337 -651.7365
## 
## Random effects:
##  Formula: ~1 | Plant
##         (Intercept)  Residual
## StdDev:   0.4836505 0.9386242
## 
## Variance function:
##  Structure: Different standard deviations per stratum
##  Formula: ~1 | Plant 
##  Parameter estimates:
##        P1        P5        P2        P7        P6        P3        P4 
## 1.0000000 0.9311479 0.7577024 1.1032408 0.9947766 0.7340284 0.9624186 
## Fixed effects:  list(fn) 
##                    Value Std.Error  DF    t-value p-value
## (Intercept)  -0.08802178 0.2063680 489 -0.4265281  0.6699
## TreatmentD04 -0.06294458 0.1305003 489 -0.4823327  0.6298
## TreatmentD07  0.08790561 0.1345620 489  0.6532720  0.5139
## TreatmentD14  0.19665690 0.1345873 489  1.4611844  0.1446
## TreatmentD21  0.19466002 0.1324728 489  1.4694334  0.1424
## TreatmentD28 -0.08417148 0.1316690 489 -0.6392656  0.5229
##  Correlation: 
##              (Intr) TrtD04 TrtD07 TrtD14 TrtD21
## TreatmentD04 -0.341                            
## TreatmentD07 -0.330  0.522                     
## TreatmentD14 -0.330  0.522  0.506              
## TreatmentD21 -0.335  0.530  0.514  0.514       
## TreatmentD28 -0.338  0.534  0.518  0.518  0.526
## 
## Standardized Within-Group Residuals:
##        Min         Q1        Med         Q3        Max 
## -2.3730826 -0.5925998 -0.1698316  0.4482653  4.5026529 
## 
## Number of Observations: 501
## Number of Groups: 7
```

```
mod_best$coefficients$random # for random intercepts
```

```
## $Plant
##    (Intercept)
## P1  -0.3203802
## P2  -0.5642745
## P3   0.2571795
## P4   0.3132635
## P5   0.8009126
## P6  -0.2230107
## P7  -0.2636901
```

Important things to interpret are the coefficient
directions/magnitude (relative to the reference category of D01; all are
scaled so as to make relative comparisons between coefficients
comparable), and the coefficients of the random intercepts are also
shown

## Model fit (R2)

```
#null model with only random intercept
mod_I_null <- lme(update(fn, .~. +1 -Treatment), 
                  data = dat, method = "REML",
                  random = ~1|Plant)

r.squaredGLMM(mod_best, null = mod_I_null) #focus on these values, below just for interest
```

```
##             R2m       R2c
## [1,] 0.01139228 0.2188065
```

```
#for comparison, fitting a full model with multiple stem parameters as predictors and seeing how this improves R2
mod_I_full <- lme(update(fn, .~. + scale(StemWidth_mm)), 
                  data = dat, method = "REML",
                  random = ~1|Plant)

r.squaredGLMM(mod_I_full, null = mod_I_null) #barely increased the R2m - clearly the biggest contributor is the plant identity to rooting variability!
```

```
##             R2m       R2c
## [1,] 0.01940198 0.2646351
```

The marginal R2 gives the model fit for the fixed effects
only (i.e. in this case, Treatment) The conditional R2 gives
the model fit that also accounts for the random effect (i.e. in this
case random intercept according to plant ID)

##Model validation

```
resids <- residuals(mod_best,type="normalized")
fitted <- fitted(mod_best)
op <- par(mfrow=c(2,2))
MyYlab <- "Residuals"
plot(x=fitted,y=resids,xlab="Fitted values",ylab=MyYlab)
qqnorm(resids)
plot(x=2*pi*dat$StemWidth_mm/2,y=resids,ylab=MyYlab,main="stem circumferance",xlab="stem circ.") 
#plot(x=dat$StemDryMass_g,y=resids,ylab=MyYlab,main="stem mass",xlab="dry mass (g)")
plot(x=as.factor(dat$Treatment),y=resids,ylab=MyYlab,main="treatment",xlab="treatment")
```

Approximately straight line in qqnorm (no glaring steps/warnings) and
residuals v. fitted are evenly distributed in variability overall and
for each of treatment and height (variability around the control
treatment is omitted now that control is removed).

Hope that this helps and makes sense?
