# Supplementary material for: Restoring South African subtropical succulent thicket using Portulacaria afra: exploring the rooting window hypothesis
Source: PeerJ. 2023 Jul 24;11:e15538. doi: 10.7717/peerj.15538 (PMC10437031; doi:10.7717/peerj.15538)
Supplement: Supplemental Information 1 — This data highlights the effects of soil moisture and parent-plant on root inititation in Portulacaria afra cuttings. [file peerj-11-15538-s001.zip › RootingWindow_Raw data and Scripts/RootingWindow_R-Script.nb.html]

rootingWindow\_PeerJ 2022


Code 

- Show All Code
- Hide All Code
- Download Rmd

# rootingWindow\_PeerJ 2022

# Load the libraries

# Get the data


```
read_csv("RootingWindow_RawData.csv")%>%
  tbl_df()->
  dat

dat%>%
  select(-DegreeOfBranching,-NoOfRootingPts)->
  dat
```

# Stem width vs stem height


```
dat%>%
  mutate(Plant="ALL")%>%
  bind_rows(dat)%>%
  filter(StemWidth_mm!=max(StemWidth_mm))%>%
  ggplot(aes(StemWidth_mm,Height_mm))+
  geom_point()+
  geom_smooth(method="lm")+
  stat_poly_line(se=FALSE) +
  stat_poly_eq(aes(label = paste(after_stat(eq.label),
                                 after_stat(rr.label), sep = "*\", \"*")))+
  facet_wrap(~Plant)+
  ylab("Height (mm)")+xlab("Stem diameter (mm)")
```

# Stem width at start of experiment vs above ground biomass at end


```
dat%>%
  mutate(Plant="ALL")%>%
  bind_rows(dat)%>%
  filter(StemWidth_mm!=max(StemWidth_mm))%>%
  ggplot(aes(StemWidth_mm,AboveGroundMass_g))+
  geom_point()+
  geom_smooth(method="lm")+
  stat_poly_line(se=FALSE) +
  stat_poly_eq(aes(label = paste(after_stat(eq.label),
                                 after_stat(rr.label), sep = "*\", \"*")))+
  facet_wrap(~Plant)+
  ylab("Above ground biomass at end of experiment")+xlab("Stem diameter (mm)")
ggsave(paste("ScatterPlot_Width_Mass_",format(Sys.time(),"%Y%m%d-%Hh%Mm"),".jpg",sep=""),width=8,height=8)
```

# Stem Height at start of experiment vs above ground biomass at end


```
dat%>%
  mutate(Plant="ALL")%>%
  bind_rows(dat)%>%
  filter(StemWidth_mm!=max(StemWidth_mm))%>%
  ggplot(aes(Height_mm,AboveGroundMass_g))+
  geom_point()+
  geom_smooth(method="lm")+
  stat_poly_line(se=FALSE) +
  stat_poly_eq(aes(label = paste(after_stat(eq.label),
                                 after_stat(rr.label), sep = "*\", \"*")))+
  facet_wrap(~Plant)+
  ylab("Above ground biomass at end of experiment")+xlab("Height (mm)")
ggsave(paste("ScatterPlot_Height_Mass_",format(Sys.time(),"%Y%m%d-%Hh%Mm"),".jpg",sep=""),width=8,height=8)
```

# Figure: Boxplot of StemWidth across Plants with post-hoc Tukey dissimilarity letters


```
dat%>%
  lm(StemWidth_mm~Plant,data=.)%>%
  aov()->anova
anova%>%
  TukeyHSD()->tukey
cld <- multcompLetters4(anova, tukey)
print(cld)
dat%>%
  group_by(Plant)%>%
  summarise(max=max(StemWidth_mm))%>%
  #summarise(mean=mean(weight), quant = quantile(weight, probs = 0.75)) %>%
  arrange(desc(max))->
  Tk
# extracting the compact letter display and adding to the Tk table
as.data.frame.list(cld$Plant)%>%
  rownames_to_column("Plant")%>%
  right_join(Tk)->
  Tk2
dat%>%
  ggplot(aes(Plant,StemWidth_mm))+
  geom_violin()+
  geom_boxplot(width=0.3)+
  geom_text(data = Tk2, aes(x = Plant, y = 10, label = Letters))+
  theme_bw()+
  xlab("Plant ID")+ylab("Basal Stem Width of Cutting (mm)")-> width.fig
width.fig
ggsave(paste("StemWidth_",format(Sys.time(),"%Y%m%d-%Hh%Mm"),".jpg",sep=""))
```

# Figure: Boxplot of Stem Height across Plants with post-hoc Tukey dissimilarity letters


```
dat%>%
  lm(Height_mm~Plant,data=.)%>%
  aov()->anova
anova%>%
  TukeyHSD()->tukey
cld <- multcompLetters4(anova, tukey)
print(cld)
dat%>%
  group_by(Plant)%>%
  summarise(max=max(Height_mm))%>%
  #summarise(mean=mean(weight), quant = quantile(weight, probs = 0.75)) %>%
  arrange(desc(max))->
  Tk
# extracting the compact letter display and adding to the Tk table
as.data.frame.list(cld$Plant)%>%
  rownames_to_column("Plant")%>%
  right_join(Tk)->
  Tk2
dat%>%
  ggplot(aes(Plant,Height_mm))+
  geom_violin()+
  geom_boxplot(width=0.3)+
  geom_text(data = Tk2, aes(x = Plant, y = 290, label = Letters))+
  theme_bw()+
  xlab("Plant ID")+ylab("Stem Height  of Cutting (mm)")-> height.fig
height.fig
#ggsave(paste("Height_",format(Sys.time(),"%Y%m%d-%Hh%Mm"),".jpg",sep=""))
```

# Figure: Boxplot of Dry Stem mass across Plants with post-hoc Tukey dissimilarity letters


```
dat%>%
  lm(StemDryMass_g~Plant,data=.)%>%
  aov()->anova
anova%>%
  TukeyHSD()->tukey
cld <- multcompLetters4(anova, tukey)
print(cld)
dat%>%
  group_by(Plant)%>%
  summarise(max=max(StemDryMass_g))%>%
  #summarise(mean=mean(weight), quant = quantile(weight, probs = 0.75)) %>%
  arrange(desc(max))->
  Tk
# extracting the compact letter display and adding to the Tk table
as.data.frame.list(cld$Plant)%>%
  rownames_to_column("Plant")%>%
  right_join(Tk)->
  Tk2
dat%>%
  ggplot(aes(Plant,StemDryMass_g))+
  geom_violin()+
  geom_boxplot(width=0.3)+
  geom_text(data = Tk2, aes(x = Plant, y = 290, label = Letters))+
  theme_bw()+
  xlab("Plant ID")+ylab("Stem Height  of Cutting (mm)")

stemdrymass.fig
#ggsave(paste("Height_",format(Sys.time(),"%Y%m%d-%Hh%Mm"),".jpg",sep=""))
```

# Combination of the two figures above


```
(width.fig+xlab("")+ggtitle('A)'))/(height.fig+xlab("")+ggtitle('B)'))
ggsave(paste("Boxplots_Width_Height_",format(Sys.time(),"%Y%m%d-%Hh%Mm"),".jpg",sep=""))
```

# Linear model and correlation of stem width with rooting fraction


```
dat%>%
  mutate(Plant="ALL")%>%
  bind_rows(dat)%>%
  ggplot(aes(StemWidth_mm,RootFraction*100))+
  geom_point()+
  geom_smooth(method="lm",se=FALSE)+
  stat_poly_line(se=FALSE) +
  stat_poly_eq(aes(label = paste(after_stat(eq.label),
                                 after_stat(rr.label), sep = "*\", \"*")))+
  facet_wrap(~Plant)+
  ylab("Root Percentage")+xlab("Stem diameter (mm)")
ggsave(paste("Width_vs_RootFraction_",format(Sys.time(),"%Y%m%d-%Hh%Mm"),".jpg",sep=""),width=8,height=8)
```

# Linear model and correlation of cutting height with rooting fraction


```
dat%>%
  mutate(Plant="ALL")%>%
  bind_rows(dat)%>%
  ggplot(aes(Height_mm,RootFraction*100))+
  geom_point()+
  geom_smooth(method="lm",se=FALSE)+
  stat_poly_line(se=FALSE) +
  stat_poly_eq(aes(label = paste(after_stat(eq.label),
                                 after_stat(rr.label), sep = "*\", \"*")))+
  facet_wrap(~Plant)+
  ylab("Root Percentage")+xlab("Plant Height (mm)")
ggsave(paste("Height_vs_RootFraction_",format(Sys.time(),"%Y%m%d-%Hh%Mm"),".jpg",sep=""),width=9,height=9)
```

# Nested ANOVA: Root mass


```
dat%>%
  filter(Treatment!="C")%>%
  filter(!is.na(RootMass_g))%>%
  aov(RootMass_g~Treatment/Plant,data=.)%>%
  summary()

dat%>%
 group_by(Plant,Treatment)%>%summarise(n=n()
                                  )
```

# Nested ANOVA: Root Fraction


```
dat%>%
  filter(Treatment!="C")%>%
  filter(!is.na(RootMass_g))%>%
  aov(RootFraction~Treatment/Plant,data=.)%>%
  summary()
```

# Root mass overall and individual boxplot


```
dat%>%
  ggplot(aes(Treatment,RootMass_g))+
  geom_boxplot(size=1,outlier.size = 2)+
  geom_boxplot(aes(fill=Plant),alpha=0.5,outlier.shape = "")+
  scale_fill_manual(values=wes_palette(7, name = "Zissou1", type = "continuous"))+
  #geom_violin()+
  #geom_line()+
  #geom_jitter(width = 0.15,aes(colour=Plant,shape=Plant))+
  #facet_wrap(~Plant)+
  ylab("Dry Root Mass (g)")
ggsave(paste("RootMassBoxplot_",format(Sys.time(),"%Y%m%d-%Hh%Mm"),".jpg",sep=""),width=7,height=5)
```

# Root mass overall and individual boxplot v2


```
dat%>%
  bind_rows(mutate(dat,Plant="All"))->
  tutu
 tutu%>%
   filter(Treatment!="C")->tutu

for (i in unique(tutu$Plant)) {
  print(i)
  tutu%>%
    filter(Plant==i)->
    toto
  kruskal.test(toto$RootMass_g,as.factor(toto$Treatment))%>%
    print()
}
 
 
res <- NULL
for (i in unique(tutu$Plant)) {
  tutu%>%
    filter(Plant==i)->
    toto
  #kruskal.test(toto$RootMass_g,as.factor(toto$Treatment))
  kruskalmc(toto$RootMass_g,as.factor(toto$Treatment)) ->
  tata
  dif3 <- tata$dif.com$difference 
  names(dif3)<-rownames(tata$dif.com)
  multcompLetters(dif3)-> tyty
  
  bind_rows(res,c(Plant=i,tyty$Letters))->res
}
# res%>%
#   mutate(C="")->res
apply(res,1,function(fx) length(unique(fx)))->ind
res[ind==2,2:7]<-""
res%>%
  pivot_longer(2:7,names_to="Treatment",values_to = "Symbol")->
  res2
tutu%>%
  group_by(Plant,Treatment)%>%
  summarise(RootMass_g=max(RootMass_g,na.rm=T))%>%
  left_join(res2)%>%
  as.data.frame()->
  sigletter

tutu%>%
  ggplot(aes(Treatment,RootMass_g))+
  geom_boxplot(size=1,outlier.size = 2)+
  #geom_boxplot(aes(fill=Plant),alpha=0.5,outlier.shape = "")+
  #scale_fill_manual(values=wes_palette(7, name = "Zissou1", type = "continuous"))+
  #geom_violin()+
  #geom_line()+
  #geom_jitter(width = 0.15,aes(colour=Plant,shape=Plant))+
  facet_wrap(~Plant,ncol=4)+
  geom_text(data=sigletter,aes(Treatment,RootMass_g+0.05,label=Symbol))+
  ylab("Dry Root Mass (g)")+
  scale_x_discrete(guide = guide_axis(n.dodge = 2)) 
ggsave(paste("RootMassBoxplot_V3_",format(Sys.time(),"%Y%m%d-%Hh%Mm"),".jpg",sep=""),width=7,height=5)
```

# Kruskal Test within plants across treatments


```
dat%>%
  filter(Treatment!="C")%>%
  group_by(Plant)%>%
  do(tidy(kruskal.test(x = .$RootMass_g, g = .$Treatment)))
```

# Rooting fraction per plant across treatments


```
(dat%>%
  filter(Treatment!="C")%>%
  group_by(Treatment,Plant)%>%
  summarise(Rooted=sum(RootMass_g>0,na.rm=T),
            n=n(),
            RootedFrac=Rooted/n*100)->for_stats)%>%
  ungroup()%>%
  ggplot(aes(Treatment,RootedFrac,label=Plant))+
  #geom_violin()+
  geom_line(colour="grey",size=3)+
  geom_text_repel(colour="darkgrey",fontface=3)+
  geom_point(size=3)+
  ylim(c(0,100))+
  ylab("Rooted percentage per plant")+
  xlab("Day of watering")+
  labs(title = expression(paste(italic("Portulacaria afra")," rooting window experiment")), 
       subtitle = "Rooting percentage per plant for day of watering during the first month\n
       Seven plants were harvested and the day of watering in the first month is shown. 12 cuttings per plant.", 
       caption = paste("Spekboom Restoration Research Group, Nelson Mandela University, 2021\n
       Figure generated by Alastair.Potts@mandela.ac.za (",Sys.time(),")",sep=""))
ggsave("RootingWindow-RootPercent.jpg",width=8,height=5)
```


```
(dat%>%
  filter(Treatment!="C")%>%
  group_by(Treatment,Plant)%>%
  summarise(Rooted=sum(RootMass_g>0,na.rm=T),
            n=n(),
            RootedFrac=Rooted/n*100)->for_stats)%>%
  ungroup()%>%
  ggplot(aes(Treatment,RootedFrac,label=Plant))+
  #geom_violin()+
  geom_line(colour="grey",size=3)+
  geom_text_repel(colour="darkgrey",fontface=3)+
  geom_point(size=3)+
  ylim(c(0,100))+
  ylab("Rooted percentage per plant")+
  xlab("Day of watering")
ggsave("Fig1_Publication_RootingWindow-RootPercent.jpg",width=7,height=4)
```

# Mean rooting fraction per plant for each treatment


```
(dat%>%
    filter(Treatment!="C")%>%
    group_by(Treatment,Plant)%>%
    summarise(Rooted=sum(RootMass_g>0,na.rm=T),
              n=n(),
              uRootMass=mean(RootMass_g,na.rm=T),
              medRootMass=median(RootMass_g)))%>%
  ungroup()%>%
  ggplot(aes(Treatment,uRootMass,label=Plant))+
  #geom_violin()+
  geom_line(colour="grey",size=3)+
  geom_text_repel(colour="darkgrey",fontface=3)+
  geom_point(size=3)+
  #ylim(c(0,100))+
  ylab("Mean root percentage per plant")+
  xlab("Day of watering")
```


```
dat%>%
    filter(Treatment!="C")%>%
    group_by(Treatment,Plant)%>%
    summarise(Rooted=sum(RootMass_g>0,na.rm=T),
              n=n(),
              uRootMass=mean(RootMass_g),
              medRootMass=median(RootMass_g,na.rm=T))%>%
  ungroup()%>%
  ggplot(aes(Treatment,medRootMass,label=Plant))+
  #geom_violin()+
  geom_line(colour="grey",size=3)+
  geom_text_repel(colour="darkgrey",fontface=3,min.segment.length = 0)+
  geom_point(size=3)+
  #ylim(c(0,100))+
  ylab("Median root percentage per plant")+
  xlab("Day of watering")
```


#Suplementary glasshouse environmental data analysis (.CSV files not
provided, if you would like to run this script please generate the
relevant data sets using “RootingWindow\_FullData” provided)


```
read_csv(construct_download_url(url,sheetid="838520867"))%>%
  tbl_df()->
  dat_temp

read_csv(construct_download_url(url,sheetid="1444740489"))%>%
  tbl_df()->
  dat_treat

dat_treat%>%
  mutate(Date=mdy_hm(paste(Date,"11h59")))->
  dat_treat2


dat_temp%>%
  mutate(Timestamp=ymd_hm(Timestamp))%>%
  ggplot(aes(Timestamp,Temperature))+
  geom_vline(xintercept = dat_treat2$Date,colour="darkgrey",size=1)+
  geom_line()+
  ylim(0,36)+
  scale_x_datetime(sec.axis = sec_axis(~.,breaks=dat_treat2$Date,labels=dat_treat2$Treatment))+
  xlab("")+
  ylab("Temperature (°C) ")+
  theme(axis.text.x.top =element_text(color="darkgrey"))+
  geom_line(aes(Timestamp,DewPoint_dC),colour="black",linetype=2)->
  A
  
dat_temp%>%
  ggplot(aes("",Temperature))+
  geom_boxplot()+
  ylim(0,36)+
  xlab("")+
  ylab("")->
  B

dat_temp%>%
  mutate(Timestamp=ymd_hm(Timestamp))%>%
  ggplot(aes(Timestamp,RelativeHumidity))+
  geom_vline(xintercept = dat_treat2$Date,colour="darkgrey",size=1)+
  geom_line()+
  ylim(0,100)+
  scale_x_datetime(sec.axis = sec_axis(~.,breaks=dat_treat2$Date,labels=dat_treat2$Treatment))+
  theme(axis.text.x.top =element_text(color="darkgrey"))+
  xlab("")+
  ylab("Relative humidity (%)")->
  C


(A+B)/C
A/C+plot_annotation(tag_levels = 'A')
ggsave("Env.conditions.jpg",width=8,height=5.5)  

dat_temp%>%
  mutate(Timestamp=ymd_hm(Timestamp),
         Day=date(Timestamp))%>%
  group_by(Day)%>%
  summarise(maxT=max(Temperature),
            minT=min(Temperature))%>%
  ungroup()%>%
  summarise(mean(maxT),
            mean(minT),
            sd(maxT),
            sd(minT))
dat_temp%>%
  summarise(mean(RelativeHumidity),sd(RelativeHumidity))
dat_temp%>%
  group_by(Da)
  summarise(uT=mean(Temperature))
```

LS0tDQp0aXRsZTogInJvb3RpbmdXaW5kb3dfUGVlckogMjAyMiINCm91dHB1dDogaHRtbF9ub3RlYm9vaw0KZWRpdG9yX29wdGlvbnM6IA0KICBjaHVua19vdXRwdXRfdHlwZTogaW5saW5lDQotLS0NCg0KIyBMb2FkIHRoZSBsaWJyYXJpZXMNCmBgYHtyIGxpYnJhcmllcywgZWNobz1GfQ0KDQpsaWJyYXJ5KCJkcGx5ciIpDQpsaWJyYXJ5KCJnZ3Bsb3QyIikNCmxpYnJhcnkoImdncG1pc2MiKQ0KbGlicmFyeSgiZ2dwdWJyIikNCmxpYnJhcnkoInBhdGNod29yayIpDQpsaWJyYXJ5KCJsdWJyaWRhdGUiKQ0KbGlicmFyeSgidGliYmxlIikNCmxpYnJhcnkoInRpZHlyIikNCmxpYnJhcnkoImdzaGVldCIpDQpsaWJyYXJ5KCJyZWFkciIpDQpsaWJyYXJ5KCJndG9vbHMiKQ0KbGlicmFyeSgid2VzYW5kZXJzb24iKQ0KbGlicmFyeSgiZ2dmb3JjZSIpDQpsaWJyYXJ5KCJnZ3JlcGVsIikNCmxpYnJhcnkoInRpYmJsZXRpbWUiKQ0KbGlicmFyeSgiYnJvb20iKQ0KbGlicmFyeSgibmxtZSIpDQpsaWJyYXJ5KCJtdWx0Y29tcFZpZXciKQ0KbGlicmFyeSgibG1lNCIpDQpsaWJyYXJ5KCJybWFya2Rvd24iKQ0KbGlicmFyeSgicGdpcm1lc3MiKQ0KDQojUGxlYXNlIHNldCB5b3VyIG93biB3b3JraW5nIGRpcmVjdG9yeQ0Kc2V0d2QoKQ0KYGBgDQoNCiMgR2V0IHRoZSBkYXRhDQpgYGB7cn0NCg0KDQpyZWFkX2NzdigiUm9vdGluZ1dpbmRvd19SYXdEYXRhLmNzdiIpJT4lDQogIHRibF9kZigpLT4NCiAgZGF0DQoNCmRhdCU+JQ0KICBzZWxlY3QoLURlZ3JlZU9mQnJhbmNoaW5nLC1Ob09mUm9vdGluZ1B0cyktPg0KICBkYXQNCg0KDQpgYGANCg0KIyBTdGVtIHdpZHRoIHZzIHN0ZW0gaGVpZ2h0DQpgYGB7cn0NCmRhdCU+JQ0KICBtdXRhdGUoUGxhbnQ9IkFMTCIpJT4lDQogIGJpbmRfcm93cyhkYXQpJT4lDQogIGZpbHRlcihTdGVtV2lkdGhfbW0hPW1heChTdGVtV2lkdGhfbW0pKSU+JQ0KICBnZ3Bsb3QoYWVzKFN0ZW1XaWR0aF9tbSxIZWlnaHRfbW0pKSsNCiAgZ2VvbV9wb2ludCgpKw0KICBnZW9tX3Ntb290aChtZXRob2Q9ImxtIikrDQogIHN0YXRfcG9seV9saW5lKHNlPUZBTFNFKSArDQogIHN0YXRfcG9seV9lcShhZXMobGFiZWwgPSBwYXN0ZShhZnRlcl9zdGF0KGVxLmxhYmVsKSwNCiAgICAgICAgICAgICAgICAgICAgICAgICAgICAgICAgIGFmdGVyX3N0YXQocnIubGFiZWwpLCBzZXAgPSAiKlwiLCBcIioiKSkpKw0KICBmYWNldF93cmFwKH5QbGFudCkrDQogIHlsYWIoIkhlaWdodCAobW0pIikreGxhYigiU3RlbSBkaWFtZXRlciAobW0pIikNCmBgYA0KDQojIFN0ZW0gd2lkdGggYXQgc3RhcnQgb2YgZXhwZXJpbWVudCB2cyBhYm92ZSBncm91bmQgYmlvbWFzcyBhdCBlbmQNCmBgYHtyfQ0KZGF0JT4lDQogIG11dGF0ZShQbGFudD0iQUxMIiklPiUNCiAgYmluZF9yb3dzKGRhdCklPiUNCiAgZmlsdGVyKFN0ZW1XaWR0aF9tbSE9bWF4KFN0ZW1XaWR0aF9tbSkpJT4lDQogIGdncGxvdChhZXMoU3RlbVdpZHRoX21tLEFib3ZlR3JvdW5kTWFzc19nKSkrDQogIGdlb21fcG9pbnQoKSsNCiAgZ2VvbV9zbW9vdGgobWV0aG9kPSJsbSIpKw0KICBzdGF0X3BvbHlfbGluZShzZT1GQUxTRSkgKw0KICBzdGF0X3BvbHlfZXEoYWVzKGxhYmVsID0gcGFzdGUoYWZ0ZXJfc3RhdChlcS5sYWJlbCksDQogICAgICAgICAgICAgICAgICAgICAgICAgICAgICAgICBhZnRlcl9zdGF0KHJyLmxhYmVsKSwgc2VwID0gIipcIiwgXCIqIikpKSsNCiAgZmFjZXRfd3JhcCh+UGxhbnQpKw0KICB5bGFiKCJBYm92ZSBncm91bmQgYmlvbWFzcyBhdCBlbmQgb2YgZXhwZXJpbWVudCIpK3hsYWIoIlN0ZW0gZGlhbWV0ZXIgKG1tKSIpDQpnZ3NhdmUocGFzdGUoIlNjYXR0ZXJQbG90X1dpZHRoX01hc3NfIixmb3JtYXQoU3lzLnRpbWUoKSwiJVklbSVkLSVIaCVNbSIpLCIuanBnIixzZXA9IiIpLHdpZHRoPTgsaGVpZ2h0PTgpDQpgYGANCiMgU3RlbSBIZWlnaHQgYXQgc3RhcnQgb2YgZXhwZXJpbWVudCB2cyBhYm92ZSBncm91bmQgYmlvbWFzcyBhdCBlbmQNCmBgYHtyfQ0KZGF0JT4lDQogIG11dGF0ZShQbGFudD0iQUxMIiklPiUNCiAgYmluZF9yb3dzKGRhdCklPiUNCiAgZmlsdGVyKFN0ZW1XaWR0aF9tbSE9bWF4KFN0ZW1XaWR0aF9tbSkpJT4lDQogIGdncGxvdChhZXMoSGVpZ2h0X21tLEFib3ZlR3JvdW5kTWFzc19nKSkrDQogIGdlb21fcG9pbnQoKSsNCiAgZ2VvbV9zbW9vdGgobWV0aG9kPSJsbSIpKw0KICBzdGF0X3BvbHlfbGluZShzZT1GQUxTRSkgKw0KICBzdGF0X3BvbHlfZXEoYWVzKGxhYmVsID0gcGFzdGUoYWZ0ZXJfc3RhdChlcS5sYWJlbCksDQogICAgICAgICAgICAgICAgICAgICAgICAgICAgICAgICBhZnRlcl9zdGF0KHJyLmxhYmVsKSwgc2VwID0gIipcIiwgXCIqIikpKSsNCiAgZmFjZXRfd3JhcCh+UGxhbnQpKw0KICB5bGFiKCJBYm92ZSBncm91bmQgYmlvbWFzcyBhdCBlbmQgb2YgZXhwZXJpbWVudCIpK3hsYWIoIkhlaWdodCAobW0pIikNCmdnc2F2ZShwYXN0ZSgiU2NhdHRlclBsb3RfSGVpZ2h0X01hc3NfIixmb3JtYXQoU3lzLnRpbWUoKSwiJVklbSVkLSVIaCVNbSIpLCIuanBnIixzZXA9IiIpLHdpZHRoPTgsaGVpZ2h0PTgpDQpgYGANCg0KIyBGaWd1cmU6IEJveHBsb3Qgb2YgU3RlbVdpZHRoIGFjcm9zcyBQbGFudHMgd2l0aCBwb3N0LWhvYyBUdWtleSBkaXNzaW1pbGFyaXR5IGxldHRlcnMNCmBgYHtyfQ0KZGF0JT4lDQogIGxtKFN0ZW1XaWR0aF9tbX5QbGFudCxkYXRhPS4pJT4lDQogIGFvdigpLT5hbm92YQ0KYW5vdmElPiUNCiAgVHVrZXlIU0QoKS0+dHVrZXkNCmNsZCA8LSBtdWx0Y29tcExldHRlcnM0KGFub3ZhLCB0dWtleSkNCnByaW50KGNsZCkNCmRhdCU+JQ0KICBncm91cF9ieShQbGFudCklPiUNCiAgc3VtbWFyaXNlKG1heD1tYXgoU3RlbVdpZHRoX21tKSklPiUNCiAgI3N1bW1hcmlzZShtZWFuPW1lYW4od2VpZ2h0KSwgcXVhbnQgPSBxdWFudGlsZSh3ZWlnaHQsIHByb2JzID0gMC43NSkpICU+JQ0KICBhcnJhbmdlKGRlc2MobWF4KSktPg0KICBUaw0KIyBleHRyYWN0aW5nIHRoZSBjb21wYWN0IGxldHRlciBkaXNwbGF5IGFuZCBhZGRpbmcgdG8gdGhlIFRrIHRhYmxlDQphcy5kYXRhLmZyYW1lLmxpc3QoY2xkJFBsYW50KSU+JQ0KICByb3duYW1lc190b19jb2x1bW4oIlBsYW50IiklPiUNCiAgcmlnaHRfam9pbihUayktPg0KICBUazINCmRhdCU+JQ0KICBnZ3Bsb3QoYWVzKFBsYW50LFN0ZW1XaWR0aF9tbSkpKw0KICBnZW9tX3Zpb2xpbigpKw0KICBnZW9tX2JveHBsb3Qod2lkdGg9MC4zKSsNCiAgZ2VvbV90ZXh0KGRhdGEgPSBUazIsIGFlcyh4ID0gUGxhbnQsIHkgPSAxMCwgbGFiZWwgPSBMZXR0ZXJzKSkrDQogIHRoZW1lX2J3KCkrDQogIHhsYWIoIlBsYW50IElEIikreWxhYigiQmFzYWwgU3RlbSBXaWR0aCBvZiBDdXR0aW5nIChtbSkiKS0+IHdpZHRoLmZpZw0Kd2lkdGguZmlnDQpnZ3NhdmUocGFzdGUoIlN0ZW1XaWR0aF8iLGZvcm1hdChTeXMudGltZSgpLCIlWSVtJWQtJUhoJU1tIiksIi5qcGciLHNlcD0iIikpDQpgYGANCg0KIyBGaWd1cmU6IEJveHBsb3Qgb2YgU3RlbSBIZWlnaHQgYWNyb3NzIFBsYW50cyB3aXRoIHBvc3QtaG9jIFR1a2V5IGRpc3NpbWlsYXJpdHkgbGV0dGVycw0KYGBge3J9DQpkYXQlPiUNCiAgbG0oSGVpZ2h0X21tflBsYW50LGRhdGE9LiklPiUNCiAgYW92KCktPmFub3ZhDQphbm92YSU+JQ0KICBUdWtleUhTRCgpLT50dWtleQ0KY2xkIDwtIG11bHRjb21wTGV0dGVyczQoYW5vdmEsIHR1a2V5KQ0KcHJpbnQoY2xkKQ0KZGF0JT4lDQogIGdyb3VwX2J5KFBsYW50KSU+JQ0KICBzdW1tYXJpc2UobWF4PW1heChIZWlnaHRfbW0pKSU+JQ0KICAjc3VtbWFyaXNlKG1lYW49bWVhbih3ZWlnaHQpLCBxdWFudCA9IHF1YW50aWxlKHdlaWdodCwgcHJvYnMgPSAwLjc1KSkgJT4lDQogIGFycmFuZ2UoZGVzYyhtYXgpKS0+DQogIFRrDQojIGV4dHJhY3RpbmcgdGhlIGNvbXBhY3QgbGV0dGVyIGRpc3BsYXkgYW5kIGFkZGluZyB0byB0aGUgVGsgdGFibGUNCmFzLmRhdGEuZnJhbWUubGlzdChjbGQkUGxhbnQpJT4lDQogIHJvd25hbWVzX3RvX2NvbHVtbigiUGxhbnQiKSU+JQ0KICByaWdodF9qb2luKFRrKS0+DQogIFRrMg0KZGF0JT4lDQogIGdncGxvdChhZXMoUGxhbnQsSGVpZ2h0X21tKSkrDQogIGdlb21fdmlvbGluKCkrDQogIGdlb21fYm94cGxvdCh3aWR0aD0wLjMpKw0KICBnZW9tX3RleHQoZGF0YSA9IFRrMiwgYWVzKHggPSBQbGFudCwgeSA9IDI5MCwgbGFiZWwgPSBMZXR0ZXJzKSkrDQogIHRoZW1lX2J3KCkrDQogIHhsYWIoIlBsYW50IElEIikreWxhYigiU3RlbSBIZWlnaHQgIG9mIEN1dHRpbmcgKG1tKSIpLT4gaGVpZ2h0LmZpZw0KaGVpZ2h0LmZpZw0KI2dnc2F2ZShwYXN0ZSgiSGVpZ2h0XyIsZm9ybWF0KFN5cy50aW1lKCksIiVZJW0lZC0lSGglTW0iKSwiLmpwZyIsc2VwPSIiKSkNCmBgYA0KDQojIEZpZ3VyZTogQm94cGxvdCBvZiBEcnkgU3RlbSBtYXNzIGFjcm9zcyBQbGFudHMgd2l0aCBwb3N0LWhvYyBUdWtleSBkaXNzaW1pbGFyaXR5IGxldHRlcnMNCmBgYHtyfQ0KZGF0JT4lDQogIGxtKFN0ZW1EcnlNYXNzX2d+UGxhbnQsZGF0YT0uKSU+JQ0KICBhb3YoKS0+YW5vdmENCmFub3ZhJT4lDQogIFR1a2V5SFNEKCktPnR1a2V5DQpjbGQgPC0gbXVsdGNvbXBMZXR0ZXJzNChhbm92YSwgdHVrZXkpDQpwcmludChjbGQpDQpkYXQlPiUNCiAgZ3JvdXBfYnkoUGxhbnQpJT4lDQogIHN1bW1hcmlzZShtYXg9bWF4KFN0ZW1EcnlNYXNzX2cpKSU+JQ0KICAjc3VtbWFyaXNlKG1lYW49bWVhbih3ZWlnaHQpLCBxdWFudCA9IHF1YW50aWxlKHdlaWdodCwgcHJvYnMgPSAwLjc1KSkgJT4lDQogIGFycmFuZ2UoZGVzYyhtYXgpKS0+DQogIFRrDQojIGV4dHJhY3RpbmcgdGhlIGNvbXBhY3QgbGV0dGVyIGRpc3BsYXkgYW5kIGFkZGluZyB0byB0aGUgVGsgdGFibGUNCmFzLmRhdGEuZnJhbWUubGlzdChjbGQkUGxhbnQpJT4lDQogIHJvd25hbWVzX3RvX2NvbHVtbigiUGxhbnQiKSU+JQ0KICByaWdodF9qb2luKFRrKS0+DQogIFRrMg0KZGF0JT4lDQogIGdncGxvdChhZXMoUGxhbnQsU3RlbURyeU1hc3NfZykpKw0KICBnZW9tX3Zpb2xpbigpKw0KICBnZW9tX2JveHBsb3Qod2lkdGg9MC4zKSsNCiAgZ2VvbV90ZXh0KGRhdGEgPSBUazIsIGFlcyh4ID0gUGxhbnQsIHkgPSAyOTAsIGxhYmVsID0gTGV0dGVycykpKw0KICB0aGVtZV9idygpKw0KICB4bGFiKCJQbGFudCBJRCIpK3lsYWIoIlN0ZW0gSGVpZ2h0ICBvZiBDdXR0aW5nIChtbSkiKQ0KDQpzdGVtZHJ5bWFzcy5maWcNCiNnZ3NhdmUocGFzdGUoIkhlaWdodF8iLGZvcm1hdChTeXMudGltZSgpLCIlWSVtJWQtJUhoJU1tIiksIi5qcGciLHNlcD0iIikpDQpgYGANCiMgQ29tYmluYXRpb24gb2YgdGhlIHR3byBmaWd1cmVzIGFib3ZlDQpgYGB7cn0NCg0KKHdpZHRoLmZpZyt4bGFiKCIiKStnZ3RpdGxlKCdBKScpKS8oaGVpZ2h0LmZpZyt4bGFiKCIiKStnZ3RpdGxlKCdCKScpKQ0KZ2dzYXZlKHBhc3RlKCJCb3hwbG90c19XaWR0aF9IZWlnaHRfIixmb3JtYXQoU3lzLnRpbWUoKSwiJVklbSVkLSVIaCVNbSIpLCIuanBnIixzZXA9IiIpKQ0KYGBgDQoNCg0KIyBMaW5lYXIgbW9kZWwgYW5kIGNvcnJlbGF0aW9uIG9mIHN0ZW0gd2lkdGggd2l0aCByb290aW5nIGZyYWN0aW9uDQpgYGB7cn0NCmRhdCU+JQ0KICBtdXRhdGUoUGxhbnQ9IkFMTCIpJT4lDQogIGJpbmRfcm93cyhkYXQpJT4lDQogIGdncGxvdChhZXMoU3RlbVdpZHRoX21tLFJvb3RGcmFjdGlvbioxMDApKSsNCiAgZ2VvbV9wb2ludCgpKw0KICBnZW9tX3Ntb290aChtZXRob2Q9ImxtIixzZT1GQUxTRSkrDQogIHN0YXRfcG9seV9saW5lKHNlPUZBTFNFKSArDQogIHN0YXRfcG9seV9lcShhZXMobGFiZWwgPSBwYXN0ZShhZnRlcl9zdGF0KGVxLmxhYmVsKSwNCiAgICAgICAgICAgICAgICAgICAgICAgICAgICAgICAgIGFmdGVyX3N0YXQocnIubGFiZWwpLCBzZXAgPSAiKlwiLCBcIioiKSkpKw0KICBmYWNldF93cmFwKH5QbGFudCkrDQogIHlsYWIoIlJvb3QgUGVyY2VudGFnZSIpK3hsYWIoIlN0ZW0gZGlhbWV0ZXIgKG1tKSIpDQpnZ3NhdmUocGFzdGUoIldpZHRoX3ZzX1Jvb3RGcmFjdGlvbl8iLGZvcm1hdChTeXMudGltZSgpLCIlWSVtJWQtJUhoJU1tIiksIi5qcGciLHNlcD0iIiksd2lkdGg9OCxoZWlnaHQ9OCkNCmBgYA0KDQoNCiMgTGluZWFyIG1vZGVsIGFuZCBjb3JyZWxhdGlvbiBvZiBjdXR0aW5nIGhlaWdodCB3aXRoIHJvb3RpbmcgZnJhY3Rpb24NCmBgYHtyfQ0KZGF0JT4lDQogIG11dGF0ZShQbGFudD0iQUxMIiklPiUNCiAgYmluZF9yb3dzKGRhdCklPiUNCiAgZ2dwbG90KGFlcyhIZWlnaHRfbW0sUm9vdEZyYWN0aW9uKjEwMCkpKw0KICBnZW9tX3BvaW50KCkrDQogIGdlb21fc21vb3RoKG1ldGhvZD0ibG0iLHNlPUZBTFNFKSsNCiAgc3RhdF9wb2x5X2xpbmUoc2U9RkFMU0UpICsNCiAgc3RhdF9wb2x5X2VxKGFlcyhsYWJlbCA9IHBhc3RlKGFmdGVyX3N0YXQoZXEubGFiZWwpLA0KICAgICAgICAgICAgICAgICAgICAgICAgICAgICAgICAgYWZ0ZXJfc3RhdChyci5sYWJlbCksIHNlcCA9ICIqXCIsIFwiKiIpKSkrDQogIGZhY2V0X3dyYXAoflBsYW50KSsNCiAgeWxhYigiUm9vdCBQZXJjZW50YWdlIikreGxhYigiUGxhbnQgSGVpZ2h0IChtbSkiKQ0KZ2dzYXZlKHBhc3RlKCJIZWlnaHRfdnNfUm9vdEZyYWN0aW9uXyIsZm9ybWF0KFN5cy50aW1lKCksIiVZJW0lZC0lSGglTW0iKSwiLmpwZyIsc2VwPSIiKSx3aWR0aD05LGhlaWdodD05KQ0KYGBgDQoNCiMgTmVzdGVkIEFOT1ZBOiBSb290IG1hc3MNCmBgYHtyfQ0KZGF0JT4lDQogIGZpbHRlcihUcmVhdG1lbnQhPSJDIiklPiUNCiAgZmlsdGVyKCFpcy5uYShSb290TWFzc19nKSklPiUNCiAgYW92KFJvb3RNYXNzX2d+VHJlYXRtZW50L1BsYW50LGRhdGE9LiklPiUNCiAgc3VtbWFyeSgpDQoNCmRhdCU+JQ0KIGdyb3VwX2J5KFBsYW50LFRyZWF0bWVudCklPiVzdW1tYXJpc2Uobj1uKCkNCiAgICAgICAgICAgICAgICAgICAgICAgICAgICAgICAgICApDQoNCmBgYA0KIyBOZXN0ZWQgQU5PVkE6IFJvb3QgRnJhY3Rpb24NCmBgYHtyfQ0KZGF0JT4lDQogIGZpbHRlcihUcmVhdG1lbnQhPSJDIiklPiUNCiAgZmlsdGVyKCFpcy5uYShSb290TWFzc19nKSklPiUNCiAgYW92KFJvb3RGcmFjdGlvbn5UcmVhdG1lbnQvUGxhbnQsZGF0YT0uKSU+JQ0KICBzdW1tYXJ5KCkNCmBgYA0KDQojIFJvb3QgbWFzcyBvdmVyYWxsIGFuZCBpbmRpdmlkdWFsIGJveHBsb3QNCmBgYHtyfQ0KZGF0JT4lDQogIGdncGxvdChhZXMoVHJlYXRtZW50LFJvb3RNYXNzX2cpKSsNCiAgZ2VvbV9ib3hwbG90KHNpemU9MSxvdXRsaWVyLnNpemUgPSAyKSsNCiAgZ2VvbV9ib3hwbG90KGFlcyhmaWxsPVBsYW50KSxhbHBoYT0wLjUsb3V0bGllci5zaGFwZSA9ICIiKSsNCiAgc2NhbGVfZmlsbF9tYW51YWwodmFsdWVzPXdlc19wYWxldHRlKDcsIG5hbWUgPSAiWmlzc291MSIsIHR5cGUgPSAiY29udGludW91cyIpKSsNCiAgI2dlb21fdmlvbGluKCkrDQogICNnZW9tX2xpbmUoKSsNCiAgI2dlb21faml0dGVyKHdpZHRoID0gMC4xNSxhZXMoY29sb3VyPVBsYW50LHNoYXBlPVBsYW50KSkrDQogICNmYWNldF93cmFwKH5QbGFudCkrDQogIHlsYWIoIkRyeSBSb290IE1hc3MgKGcpIikNCmdnc2F2ZShwYXN0ZSgiUm9vdE1hc3NCb3hwbG90XyIsZm9ybWF0KFN5cy50aW1lKCksIiVZJW0lZC0lSGglTW0iKSwiLmpwZyIsc2VwPSIiKSx3aWR0aD03LGhlaWdodD01KQ0KYGBgDQojIFJvb3QgbWFzcyBvdmVyYWxsIGFuZCBpbmRpdmlkdWFsIGJveHBsb3QgdjINCmBgYHtyfQ0KZGF0JT4lDQogIGJpbmRfcm93cyhtdXRhdGUoZGF0LFBsYW50PSJBbGwiKSktPg0KICB0dXR1DQogdHV0dSU+JQ0KICAgZmlsdGVyKFRyZWF0bWVudCE9IkMiKS0+dHV0dQ0KDQpmb3IgKGkgaW4gdW5pcXVlKHR1dHUkUGxhbnQpKSB7DQogIHByaW50KGkpDQogIHR1dHUlPiUNCiAgICBmaWx0ZXIoUGxhbnQ9PWkpLT4NCiAgICB0b3RvDQogIGtydXNrYWwudGVzdCh0b3RvJFJvb3RNYXNzX2csYXMuZmFjdG9yKHRvdG8kVHJlYXRtZW50KSklPiUNCiAgICBwcmludCgpDQp9DQogDQogDQpyZXMgPC0gTlVMTA0KZm9yIChpIGluIHVuaXF1ZSh0dXR1JFBsYW50KSkgew0KICB0dXR1JT4lDQogICAgZmlsdGVyKFBsYW50PT1pKS0+DQogICAgdG90bw0KICAja3J1c2thbC50ZXN0KHRvdG8kUm9vdE1hc3NfZyxhcy5mYWN0b3IodG90byRUcmVhdG1lbnQpKQ0KICBrcnVza2FsbWModG90byRSb290TWFzc19nLGFzLmZhY3Rvcih0b3RvJFRyZWF0bWVudCkpIC0+DQogIHRhdGENCiAgZGlmMyA8LSB0YXRhJGRpZi5jb20kZGlmZmVyZW5jZSANCiAgbmFtZXMoZGlmMyk8LXJvd25hbWVzKHRhdGEkZGlmLmNvbSkNCiAgbXVsdGNvbXBMZXR0ZXJzKGRpZjMpLT4gdHl0eQ0KICANCiAgYmluZF9yb3dzKHJlcyxjKFBsYW50PWksdHl0eSRMZXR0ZXJzKSktPnJlcw0KfQ0KIyByZXMlPiUNCiMgICBtdXRhdGUoQz0iIiktPnJlcw0KYXBwbHkocmVzLDEsZnVuY3Rpb24oZngpIGxlbmd0aCh1bmlxdWUoZngpKSktPmluZA0KcmVzW2luZD09MiwyOjddPC0iIg0KcmVzJT4lDQogIHBpdm90X2xvbmdlcigyOjcsbmFtZXNfdG89IlRyZWF0bWVudCIsdmFsdWVzX3RvID0gIlN5bWJvbCIpLT4NCiAgcmVzMg0KdHV0dSU+JQ0KICBncm91cF9ieShQbGFudCxUcmVhdG1lbnQpJT4lDQogIHN1bW1hcmlzZShSb290TWFzc19nPW1heChSb290TWFzc19nLG5hLnJtPVQpKSU+JQ0KICBsZWZ0X2pvaW4ocmVzMiklPiUNCiAgYXMuZGF0YS5mcmFtZSgpLT4NCiAgc2lnbGV0dGVyDQoNCnR1dHUlPiUNCiAgZ2dwbG90KGFlcyhUcmVhdG1lbnQsUm9vdE1hc3NfZykpKw0KICBnZW9tX2JveHBsb3Qoc2l6ZT0xLG91dGxpZXIuc2l6ZSA9IDIpKw0KICAjZ2VvbV9ib3hwbG90KGFlcyhmaWxsPVBsYW50KSxhbHBoYT0wLjUsb3V0bGllci5zaGFwZSA9ICIiKSsNCiAgI3NjYWxlX2ZpbGxfbWFudWFsKHZhbHVlcz13ZXNfcGFsZXR0ZSg3LCBuYW1lID0gIlppc3NvdTEiLCB0eXBlID0gImNvbnRpbnVvdXMiKSkrDQogICNnZW9tX3Zpb2xpbigpKw0KICAjZ2VvbV9saW5lKCkrDQogICNnZW9tX2ppdHRlcih3aWR0aCA9IDAuMTUsYWVzKGNvbG91cj1QbGFudCxzaGFwZT1QbGFudCkpKw0KICBmYWNldF93cmFwKH5QbGFudCxuY29sPTQpKw0KICBnZW9tX3RleHQoZGF0YT1zaWdsZXR0ZXIsYWVzKFRyZWF0bWVudCxSb290TWFzc19nKzAuMDUsbGFiZWw9U3ltYm9sKSkrDQogIHlsYWIoIkRyeSBSb290IE1hc3MgKGcpIikrDQogIHNjYWxlX3hfZGlzY3JldGUoZ3VpZGUgPSBndWlkZV9heGlzKG4uZG9kZ2UgPSAyKSkgDQpnZ3NhdmUocGFzdGUoIlJvb3RNYXNzQm94cGxvdF9WM18iLGZvcm1hdChTeXMudGltZSgpLCIlWSVtJWQtJUhoJU1tIiksIi5qcGciLHNlcD0iIiksd2lkdGg9NyxoZWlnaHQ9NSkNCmBgYA0KDQoNCiMgS3J1c2thbCBUZXN0IHdpdGhpbiBwbGFudHMgYWNyb3NzIHRyZWF0bWVudHMNCmBgYHtyfQ0KZGF0JT4lDQogIGZpbHRlcihUcmVhdG1lbnQhPSJDIiklPiUNCiAgZ3JvdXBfYnkoUGxhbnQpJT4lDQogIGRvKHRpZHkoa3J1c2thbC50ZXN0KHggPSAuJFJvb3RNYXNzX2csIGcgPSAuJFRyZWF0bWVudCkpKQ0KYGBgDQoNCiMgUm9vdGluZyBmcmFjdGlvbiBwZXIgcGxhbnQgYWNyb3NzIHRyZWF0bWVudHMNCmBgYHtyfQ0KKGRhdCU+JQ0KICBmaWx0ZXIoVHJlYXRtZW50IT0iQyIpJT4lDQogIGdyb3VwX2J5KFRyZWF0bWVudCxQbGFudCklPiUNCiAgc3VtbWFyaXNlKFJvb3RlZD1zdW0oUm9vdE1hc3NfZz4wLG5hLnJtPVQpLA0KICAgICAgICAgICAgbj1uKCksDQogICAgICAgICAgICBSb290ZWRGcmFjPVJvb3RlZC9uKjEwMCktPmZvcl9zdGF0cyklPiUNCiAgdW5ncm91cCgpJT4lDQogIGdncGxvdChhZXMoVHJlYXRtZW50LFJvb3RlZEZyYWMsbGFiZWw9UGxhbnQpKSsNCiAgI2dlb21fdmlvbGluKCkrDQogIGdlb21fbGluZShjb2xvdXI9ImdyZXkiLHNpemU9MykrDQogIGdlb21fdGV4dF9yZXBlbChjb2xvdXI9ImRhcmtncmV5Iixmb250ZmFjZT0zKSsNCiAgZ2VvbV9wb2ludChzaXplPTMpKw0KICB5bGltKGMoMCwxMDApKSsNCiAgeWxhYigiUm9vdGVkIHBlcmNlbnRhZ2UgcGVyIHBsYW50IikrDQogIHhsYWIoIkRheSBvZiB3YXRlcmluZyIpKw0KICBsYWJzKHRpdGxlID0gZXhwcmVzc2lvbihwYXN0ZShpdGFsaWMoIlBvcnR1bGFjYXJpYSBhZnJhIiksIiByb290aW5nIHdpbmRvdyBleHBlcmltZW50IikpLCANCiAgICAgICBzdWJ0aXRsZSA9ICJSb290aW5nIHBlcmNlbnRhZ2UgcGVyIHBsYW50IGZvciBkYXkgb2Ygd2F0ZXJpbmcgZHVyaW5nIHRoZSBmaXJzdCBtb250aFxuDQogICAgICAgU2V2ZW4gcGxhbnRzIHdlcmUgaGFydmVzdGVkIGFuZCB0aGUgZGF5IG9mIHdhdGVyaW5nIGluIHRoZSBmaXJzdCBtb250aCBpcyBzaG93bi4gMTIgY3V0dGluZ3MgcGVyIHBsYW50LiIsIA0KICAgICAgIGNhcHRpb24gPSBwYXN0ZSgiU3Bla2Jvb20gUmVzdG9yYXRpb24gUmVzZWFyY2ggR3JvdXAsIE5lbHNvbiBNYW5kZWxhIFVuaXZlcnNpdHksIDIwMjFcbg0KICAgICAgIEZpZ3VyZSBnZW5lcmF0ZWQgYnkgQWxhc3RhaXIuUG90dHNAbWFuZGVsYS5hYy56YSAoIixTeXMudGltZSgpLCIpIixzZXA9IiIpKQ0KZ2dzYXZlKCJSb290aW5nV2luZG93LVJvb3RQZXJjZW50LmpwZyIsd2lkdGg9OCxoZWlnaHQ9NSkNCmBgYA0KDQpgYGB7cn0NCihkYXQlPiUNCiAgZmlsdGVyKFRyZWF0bWVudCE9IkMiKSU+JQ0KICBncm91cF9ieShUcmVhdG1lbnQsUGxhbnQpJT4lDQogIHN1bW1hcmlzZShSb290ZWQ9c3VtKFJvb3RNYXNzX2c+MCxuYS5ybT1UKSwNCiAgICAgICAgICAgIG49bigpLA0KICAgICAgICAgICAgUm9vdGVkRnJhYz1Sb290ZWQvbioxMDApLT5mb3Jfc3RhdHMpJT4lDQogIHVuZ3JvdXAoKSU+JQ0KICBnZ3Bsb3QoYWVzKFRyZWF0bWVudCxSb290ZWRGcmFjLGxhYmVsPVBsYW50KSkrDQogICNnZW9tX3Zpb2xpbigpKw0KICBnZW9tX2xpbmUoY29sb3VyPSJncmV5IixzaXplPTMpKw0KICBnZW9tX3RleHRfcmVwZWwoY29sb3VyPSJkYXJrZ3JleSIsZm9udGZhY2U9MykrDQogIGdlb21fcG9pbnQoc2l6ZT0zKSsNCiAgeWxpbShjKDAsMTAwKSkrDQogIHlsYWIoIlJvb3RlZCBwZXJjZW50YWdlIHBlciBwbGFudCIpKw0KICB4bGFiKCJEYXkgb2Ygd2F0ZXJpbmciKQ0KZ2dzYXZlKCJGaWcxX1B1YmxpY2F0aW9uX1Jvb3RpbmdXaW5kb3ctUm9vdFBlcmNlbnQuanBnIix3aWR0aD03LGhlaWdodD00KQ0KYGBgDQoNCg0KIyBNZWFuIHJvb3RpbmcgZnJhY3Rpb24gcGVyIHBsYW50IGZvciBlYWNoIHRyZWF0bWVudA0KYGBge3J9DQooZGF0JT4lDQogICAgZmlsdGVyKFRyZWF0bWVudCE9IkMiKSU+JQ0KICAgIGdyb3VwX2J5KFRyZWF0bWVudCxQbGFudCklPiUNCiAgICBzdW1tYXJpc2UoUm9vdGVkPXN1bShSb290TWFzc19nPjAsbmEucm09VCksDQogICAgICAgICAgICAgIG49bigpLA0KICAgICAgICAgICAgICB1Um9vdE1hc3M9bWVhbihSb290TWFzc19nLG5hLnJtPVQpLA0KICAgICAgICAgICAgICBtZWRSb290TWFzcz1tZWRpYW4oUm9vdE1hc3NfZykpKSU+JQ0KICB1bmdyb3VwKCklPiUNCiAgZ2dwbG90KGFlcyhUcmVhdG1lbnQsdVJvb3RNYXNzLGxhYmVsPVBsYW50KSkrDQogICNnZW9tX3Zpb2xpbigpKw0KICBnZW9tX2xpbmUoY29sb3VyPSJncmV5IixzaXplPTMpKw0KICBnZW9tX3RleHRfcmVwZWwoY29sb3VyPSJkYXJrZ3JleSIsZm9udGZhY2U9MykrDQogIGdlb21fcG9pbnQoc2l6ZT0zKSsNCiAgI3lsaW0oYygwLDEwMCkpKw0KICB5bGFiKCJNZWFuIHJvb3QgcGVyY2VudGFnZSBwZXIgcGxhbnQiKSsNCiAgeGxhYigiRGF5IG9mIHdhdGVyaW5nIikgIA0KYGBgDQoNCmBgYHtyfQ0KZGF0JT4lDQogICAgZmlsdGVyKFRyZWF0bWVudCE9IkMiKSU+JQ0KICAgIGdyb3VwX2J5KFRyZWF0bWVudCxQbGFudCklPiUNCiAgICBzdW1tYXJpc2UoUm9vdGVkPXN1bShSb290TWFzc19nPjAsbmEucm09VCksDQogICAgICAgICAgICAgIG49bigpLA0KICAgICAgICAgICAgICB1Um9vdE1hc3M9bWVhbihSb290TWFzc19nKSwNCiAgICAgICAgICAgICAgbWVkUm9vdE1hc3M9bWVkaWFuKFJvb3RNYXNzX2csbmEucm09VCkpJT4lDQogIHVuZ3JvdXAoKSU+JQ0KICBnZ3Bsb3QoYWVzKFRyZWF0bWVudCxtZWRSb290TWFzcyxsYWJlbD1QbGFudCkpKw0KICAjZ2VvbV92aW9saW4oKSsNCiAgZ2VvbV9saW5lKGNvbG91cj0iZ3JleSIsc2l6ZT0zKSsNCiAgZ2VvbV90ZXh0X3JlcGVsKGNvbG91cj0iZGFya2dyZXkiLGZvbnRmYWNlPTMsbWluLnNlZ21lbnQubGVuZ3RoID0gMCkrDQogIGdlb21fcG9pbnQoc2l6ZT0zKSsNCiAgI3lsaW0oYygwLDEwMCkpKw0KICB5bGFiKCJNZWRpYW4gcm9vdCBwZXJjZW50YWdlIHBlciBwbGFudCIpKw0KICB4bGFiKCJEYXkgb2Ygd2F0ZXJpbmciKSAgDQpgYGANCg0KI1N1cGxlbWVudGFyeSBnbGFzc2hvdXNlIGVudmlyb25tZW50YWwgZGF0YSBhbmFseXNpcyAoLkNTViBmaWxlcyBub3QgcHJvdmlkZWQsIGlmIHlvdSB3b3VsZCBsaWtlIHRvIHJ1biB0aGlzIHNjcmlwdCBwbGVhc2UgZ2VuZXJhdGUgdGhlIHJlbGV2YW50IGRhdGEgc2V0cyB1c2luZyAiUm9vdGluZ1dpbmRvd19GdWxsRGF0YSIgcHJvdmlkZWQpDQpgYGB7cn0NCnJlYWRfY3N2KGNvbnN0cnVjdF9kb3dubG9hZF91cmwodXJsLHNoZWV0aWQ9IjgzODUyMDg2NyIpKSU+JQ0KICB0YmxfZGYoKS0+DQogIGRhdF90ZW1wDQoNCnJlYWRfY3N2KGNvbnN0cnVjdF9kb3dubG9hZF91cmwodXJsLHNoZWV0aWQ9IjE0NDQ3NDA0ODkiKSklPiUNCiAgdGJsX2RmKCktPg0KICBkYXRfdHJlYXQNCg0KZGF0X3RyZWF0JT4lDQogIG11dGF0ZShEYXRlPW1keV9obShwYXN0ZShEYXRlLCIxMWg1OSIpKSktPg0KICBkYXRfdHJlYXQyDQoNCg0KDQpkYXRfdGVtcCU+JQ0KICBtdXRhdGUoVGltZXN0YW1wPXltZF9obShUaW1lc3RhbXApKSU+JQ0KICBnZ3Bsb3QoYWVzKFRpbWVzdGFtcCxUZW1wZXJhdHVyZSkpKw0KICBnZW9tX3ZsaW5lKHhpbnRlcmNlcHQgPSBkYXRfdHJlYXQyJERhdGUsY29sb3VyPSJkYXJrZ3JleSIsc2l6ZT0xKSsNCiAgZ2VvbV9saW5lKCkrDQogIHlsaW0oMCwzNikrDQogIHNjYWxlX3hfZGF0ZXRpbWUoc2VjLmF4aXMgPSBzZWNfYXhpcyh+LixicmVha3M9ZGF0X3RyZWF0MiREYXRlLGxhYmVscz1kYXRfdHJlYXQyJFRyZWF0bWVudCkpKw0KICB4bGFiKCIiKSsNCiAgeWxhYigiVGVtcGVyYXR1cmUgKMKwQykgIikrDQogIHRoZW1lKGF4aXMudGV4dC54LnRvcCA9ZWxlbWVudF90ZXh0KGNvbG9yPSJkYXJrZ3JleSIpKSsNCiAgZ2VvbV9saW5lKGFlcyhUaW1lc3RhbXAsRGV3UG9pbnRfZEMpLGNvbG91cj0iYmxhY2siLGxpbmV0eXBlPTIpLT4NCiAgQQ0KICANCmRhdF90ZW1wJT4lDQogIGdncGxvdChhZXMoIiIsVGVtcGVyYXR1cmUpKSsNCiAgZ2VvbV9ib3hwbG90KCkrDQogIHlsaW0oMCwzNikrDQogIHhsYWIoIiIpKw0KICB5bGFiKCIiKS0+DQogIEINCg0KZGF0X3RlbXAlPiUNCiAgbXV0YXRlKFRpbWVzdGFtcD15bWRfaG0oVGltZXN0YW1wKSklPiUNCiAgZ2dwbG90KGFlcyhUaW1lc3RhbXAsUmVsYXRpdmVIdW1pZGl0eSkpKw0KICBnZW9tX3ZsaW5lKHhpbnRlcmNlcHQgPSBkYXRfdHJlYXQyJERhdGUsY29sb3VyPSJkYXJrZ3JleSIsc2l6ZT0xKSsNCiAgZ2VvbV9saW5lKCkrDQogIHlsaW0oMCwxMDApKw0KICBzY2FsZV94X2RhdGV0aW1lKHNlYy5heGlzID0gc2VjX2F4aXMofi4sYnJlYWtzPWRhdF90cmVhdDIkRGF0ZSxsYWJlbHM9ZGF0X3RyZWF0MiRUcmVhdG1lbnQpKSsNCiAgdGhlbWUoYXhpcy50ZXh0LngudG9wID1lbGVtZW50X3RleHQoY29sb3I9ImRhcmtncmV5IikpKw0KICB4bGFiKCIiKSsNCiAgeWxhYigiUmVsYXRpdmUgaHVtaWRpdHkgKCUpIiktPg0KICBDDQoNCg0KKEErQikvQw0KQS9DK3Bsb3RfYW5ub3RhdGlvbih0YWdfbGV2ZWxzID0gJ0EnKQ0KZ2dzYXZlKCJFbnYuY29uZGl0aW9ucy5qcGciLHdpZHRoPTgsaGVpZ2h0PTUuNSkgIA0KDQpkYXRfdGVtcCU+JQ0KICBtdXRhdGUoVGltZXN0YW1wPXltZF9obShUaW1lc3RhbXApLA0KICAgICAgICAgRGF5PWRhdGUoVGltZXN0YW1wKSklPiUNCiAgZ3JvdXBfYnkoRGF5KSU+JQ0KICBzdW1tYXJpc2UobWF4VD1tYXgoVGVtcGVyYXR1cmUpLA0KICAgICAgICAgICAgbWluVD1taW4oVGVtcGVyYXR1cmUpKSU+JQ0KICB1bmdyb3VwKCklPiUNCiAgc3VtbWFyaXNlKG1lYW4obWF4VCksDQogICAgICAgICAgICBtZWFuKG1pblQpLA0KICAgICAgICAgICAgc2QobWF4VCksDQogICAgICAgICAgICBzZChtaW5UKSkNCmRhdF90ZW1wJT4lDQogIHN1bW1hcmlzZShtZWFuKFJlbGF0aXZlSHVtaWRpdHkpLHNkKFJlbGF0aXZlSHVtaWRpdHkpKQ0KZGF0X3RlbXAlPiUNCiAgZ3JvdXBfYnkoRGEpDQogIHN1bW1hcmlzZSh1VD1tZWFuKFRlbXBlcmF0dXJlKSkNCg0KYGBgDQoNCg==
