# Supplementary material for: Restoring South African subtropical succulent thicket using Portulacaria afra: exploring the rooting window hypothesis
Source: PeerJ. 2023 Jul 24;11:e15538. doi: 10.7717/peerj.15538 (PMC10437031; doi:10.7717/peerj.15538)
Supplement: Supplemental Information 2 [file peerj-11-15538-s002.doc]

| Dataset | df | stat | p |
| --- | --- | --- | --- |
| All | 5 | 9,7191 | 0,083 |
| P1 | 5 | 25,192 | 0,0001 |
| P2 | 5 | 9,4676 | 0,092 |
| P3 | 5 | 1,3771 | 0,927 |
| P4 | 5 | 4,3791 | 0,496 |
| P5 | 5 | 14,649 | 0,012 |
| P6 | 5 | 7,2458 | 0,203 |
| P7 | 5 | 11,247 | 0,047 |
