# Supplementary figures and images for: Restoring South African subtropical succulent thicket using Portulacaria afra: exploring the rooting window hypothesis
Source: PeerJ. 2023 Jul 24;11:e15538. doi: 10.7717/peerj.15538 (PMC10437031; doi:10.7717/peerj.15538)

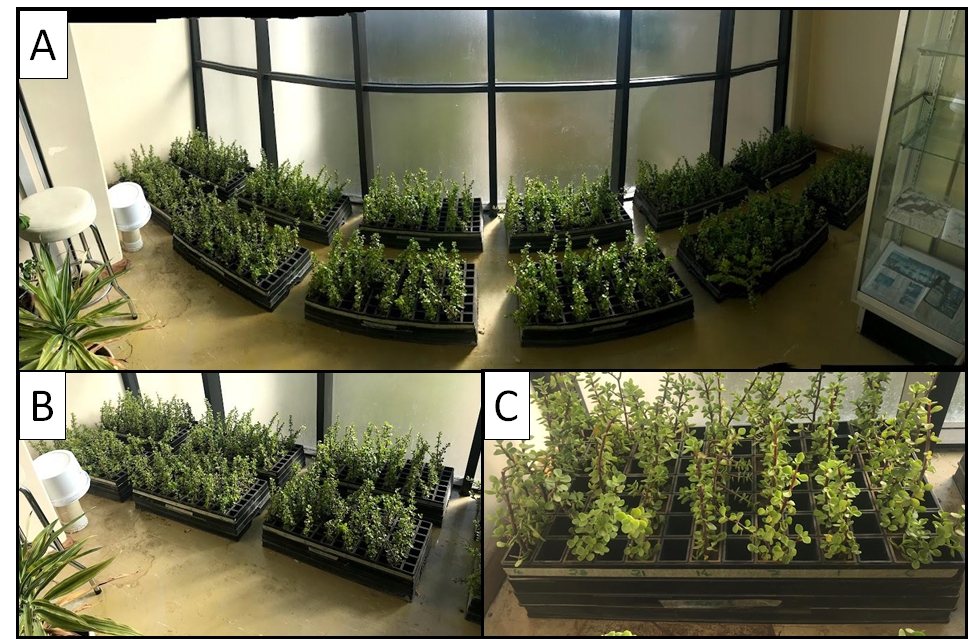

Supplement: Supplemental Information 3 [file peerj-11-15538-s003.jpg]

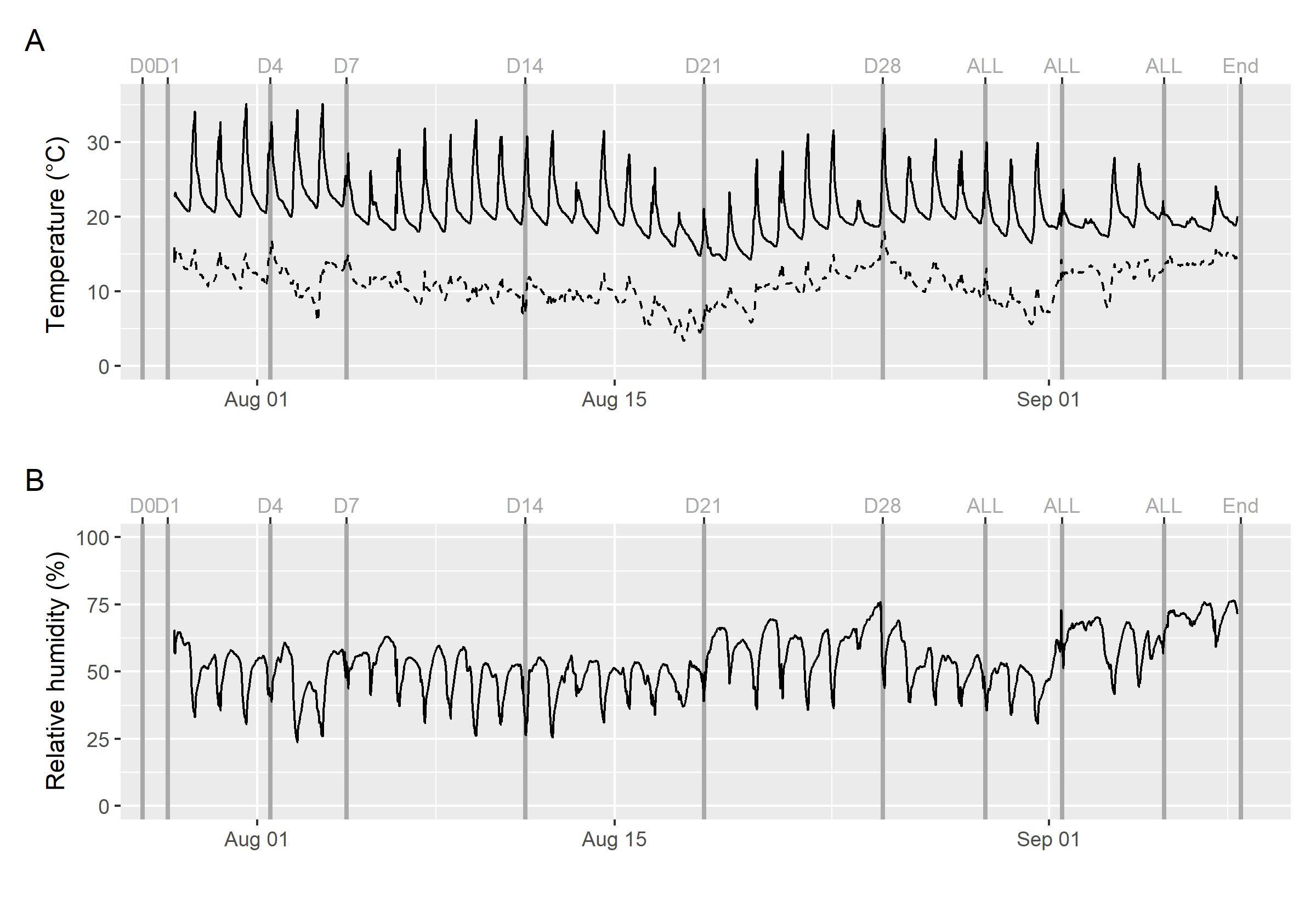

Supplement: Supplemental Information 4 — This data was collected by a Hairuis SSN-22 USB temperature humidity logger (Banao Area, Shenzhen China). [file peerj-11-15538-s004.jpg]
